# Histone deacetylase HDAC3 regulates ergosterol production for oxidative stress tolerance in the entomopathogenic and endophytic fungus *Metarhizium robertsii*

**DOI:** 10.1128/msystems.00953-24

**Published:** 2024-09-17

**Authors:** Shuxing Liu, Xinmiao Wang, Xingyuan Tang, Weiguo Fang

**Affiliations:** 1 MOE Key Laboratory of Biosystems Homeostasis and Protection, Institute of Microbiology, College of Life Science, Zhejiang University, Hangzhou, China; University of Toronto, Toronto, Ontario, Canada

**Keywords:** entomopathogenic fungi, plant symbiotic fungi, *Metarhizium*, epigenetic, histone deacetylase, pathogenicity, symbiosis, ergosterol, oxidative stress

## Abstract

Oxidative stress is encountered by fungi in almost all niches, resulting in fungal degeneration or even death. Fungal tolerance to oxidative stress has been extensively studied, but the current understanding of the mechanisms regulating oxidative stress tolerance in fungi remains limited. The entomopathogenic and endophytic fungus *Metarhizium robertsii* encounters oxidative stress when it infects insects and develops a symbiotic relationship with plants, and we found that host reactive oxygen species (ROSs) greatly limited fungal growth in both insects and plants. We identified a histone H3 deacetylase (HDAC3) that catalyzed the deacetylation of lysine 56 of histone H3. Deleting *Hdac3* significantly reduced the tolerance of *M. robertsii* to oxidative stress from insects and plants, thereby decreasing fungal ability to colonize the insect hemocoel and plant roots. HDAC3 achieved this by regulating the expression of three genes in the ergosterol biosynthesis pathway, which includes the lanosterol synthase gene *Las1*. The deletion of *Hdac3* or *Las1* reduced the ergosterol content and impaired cell membrane integrity. This resulted in an increase in ROS accumulation in fungal cells that were thus more sensitive to oxidative stress. We further showed that HDAC3 regulated the expression of the three ergosterol biosynthesis genes in an indirect manner. Our work significantly advances insights into the epigenetic regulation of oxidative stress tolerance and the interactions between *M. robertsii* and its plant and insect hosts.

**IMPORTANCE:**

Oxidative stress is a common challenge encountered by fungi that have evolved sophisticated mechanisms underlying tolerance to this stress. Although fungal tolerance to oxidative stress has been extensively investigated, the current understanding of the mechanisms for fungi to regulate oxidative stress tolerance remains limited. In the model entomopathogenic and plant symbiotic fungus *Metarhizium robertsii*, we found that the histone H3 deacetylase HDAC3 regulates the production of ergosterol, an essential cell membrane component. This maintains the cell membrane integrity to resist the oxidative stress derived from the insect and plant hosts for successful infection of insects and development of symbiotic associates with plants. Our work provides significant insights into the regulation of oxidative stress tolerance in *M. robertsii* and its interactions with insects and plants.

## INTRODUCTION

*Metarhizium robertsii* is a versatile fungus with saprophytic, plant symbiotic, and entomopathogenic lifestyle options and is used as a representative for studying broad themes of switch among multiple lifestyles (1). To establish different relationships with multiple hosts, *M. robertsii* encounters diverse microenvironments. Infection of a susceptible insect host occurs when conidia adhere to the cuticle and produce germ tubes for cuticle penetration. Once reaching the insect hemocoel, the fungus undergoes dimorphism from hyphae to yeast-like cells (i.e., hyphal bodies), which proliferate in the insect hemocoel for successful colonization (2). *M. robertsii* thus encounters two distinctive microenvironments (the cuticle and the hemocoel) to establish a parasitic relationship with insects (2). To develop a symbiotic relationship with plants, *M. robertsii* also encounters multiple microenvironments including the rhizosphere, rhizoplane, and plant tissues (3, 4).

Survival in several different environments requires fine-tuning regulation of gene expression programs. So far, knowledge of regulatory mechanisms underlying the interaction of *Metarhizium* with plants remains limited, while regulation of infection of insects has been extensively investigated. Precise regulation of the membrane protein Mr-OPY2 and its downstream transcription factor AFTF1 is essential for the initiation of cuticle penetration (1). The Myb transcription factor RNS1, a central regulator, channels information from the Fus3- and Slt2-MAPK cascade to activate the penetration of the cuticle (5, 6). G-protein-coupled receptors were also important for host recognition and appressorial formation (7).

Epigenetic regulation of gene expression is achieved by histone modifications and non-coding RNAs-associated gene silencing, and it plays crucial roles in the fungal growth and development, and production of secondary metabolites (8). However, epigenetic regulation has been much less explored in *M. robertsii* with only several epigenetic regulators reported to control infection of insects. In the insect hemocoel, repression conferred by the histone deacetylase HDAC1 and the histone 3 acetyltransferase HAT1 is reduced, inducing the expression of the regulatory protein COH1. COH1 interacts with transcription factor COH2 to reduce COH2 stability, thereby downregulating cuticle penetration genes and upregulating genes for hemocoel colonization (2). In addition, DNA methyltransferase MrRID and MrDIM-2 (9), as well as histone lysine methyltransferase KMT2 (10) and ASH1 (11), have also been reported to regulate fungal pathogenicity. In this study, we screened a library of epigenetic regulator mutants to identify new regulators and elucidate epigenetic regulation of infection of insects by *M. robertsii*. We found that the histone H3 deacetylase HDAC3, via regulation of ergosterol production for tolerance to oxidative stress, not only regulated the infection of insects but also the development of the symbiotic relationship with plants.

## RESULTS

### Identification of the histone H3 deacetylase HDAC3

**Fig 1:**
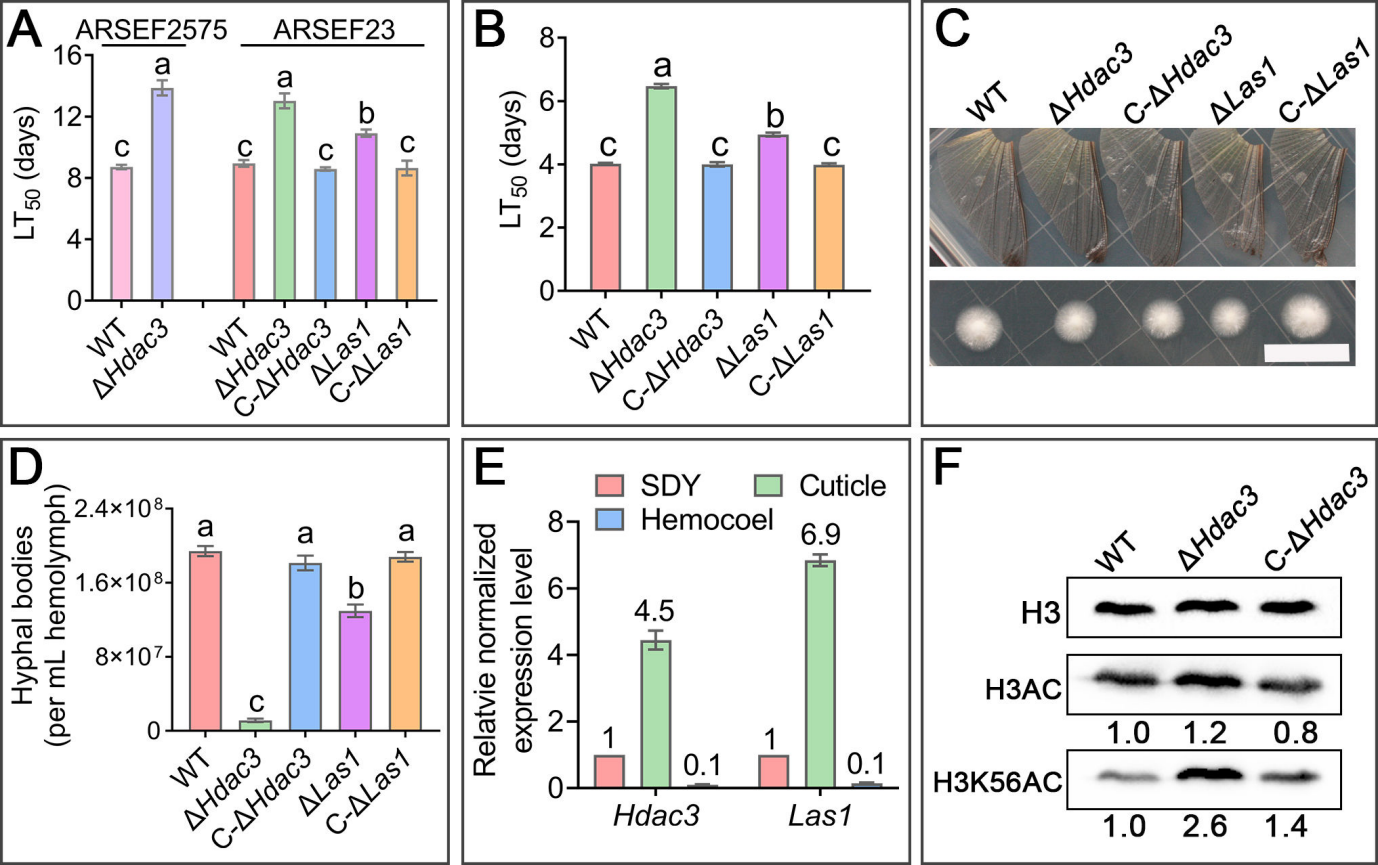
Pathogenicity of the WT, Δ*Hdac3,* and Δ*Las1*, and the complemented strains C-Δ*Hdac3* and C-Δ*Las1*. (**A**) LT_50_ (median lethal time: time taken to kill 50% of insects) values via topical application of spores on *G. mellonella* larvae. (**B**) LT_50_ values via direct injection of ARSEF23 spores into the insect hemocoel. Except for the data shown in Fig. 1A, all other data are about the strain ARSEF23. Data are shown as the means ± SE, which is applicable to all LT_50_ values in this study. Values with different letters are significantly different (*P* < 0.05, Tukey’s test in one-way ANOVA). (**C**) Penetration of locust hindwings. Spores were inoculated on the cuticle placed on potato dextrose agar (PDA) plates for penetration at 26°C for 48 h (upper panel). The hindwings were then removed, and the plates were further incubated for 48 h to allow fungal growth (lower panel). Scale bar: 2 cm. Images are representative of three independent experiments. (**D**) The number of hyphal bodies in the hemolymph collected from the *G. mellonella* larvae inoculated via direct injection. Data are shown as the means ± SE. Values with different letters are significantly different (*P* < 0.05, Tukey’s test in one-way ANOVA). (**E**) qRT-PCR analysis of the expression of *Hdac3* and *Las1* on the cuticle of *G. mellonella* larvae (Cuticle) and in the insect hemocoel (Hemocoel) relative to the saprophytic growth in the Saubraud dextrose broth supplemented with 1% of yeast extract medium (SDY). This analysis was repeated three times with three replicates; the values for a gene show the fold changes in the expression of the gene in an environment compared with SDY, which is set to 1. Data are shown as the means ± SE. This legend is applicable to all other qRT-PCR analyses conducted in this study. (**F**) Immunoblot analysis showing HDAC3 regulated acetylation level of histone H3 on lysine 56 (H3K56AC). H3, histone H3; H3AC, acetylation level of histone H3. Images shown are representatives of at least three independent experiments. Numbers indicate the band intensity for acetylation level of histone H3 (lysine 56 in histone H3) relative to histone H3.

To investigate the epigenetic regulation of infection of insects by *M. robertsii*, we first tried to identify epigenetic regulators by analyzing the pathogenicity of a series of previously constructed mutants of the *M. robertsii* strain ARSEF2575 each with an epigenetic regulator encoding gene deleted (2). We found a mutant of a putative NAD^+^-dependent histone H3 deacetylase gene (GenBank accession number: MAA_04246, designated as *Hdac3*) showed impaired virulence against the great wax moth larvae (*Galleria mellonella*) (Fig. 1A). HDAC3 is a Sir2 (silent information regulator 2) family protein with a conserved domain (PFAM02416).

Since *Hdac3* regulated the proliferation of hyphal bodies in the hemocoel (see below) and the *M. robertsii* strain ARSEF2575 produced very few hyphal bodies (2), the *M. robertsii* ARSEF23 strain, which produces many hyphal bodies in the hemocoel (12), was thus used to assay how *Hdac3* regulates the proliferation of hyphal bodies. The *Hdac3* deletion mutant Δ*Hdac3* of *M. robertsii* ARSEF23 was thus constructed, which was then complemented with the wild-type (WT) gene of *Hdac3* to produce C-Δ*Hdac3* (Fig. S1A through C). Bioassays showed that the rate Δ*Hdac3* killed *G. mellonella* larvae was 1.5-fold slower than the WT via topical application of spores on the cuticle (1.6-fold through direct hemocoel injection) (*P* < 0.05) (Fig. 1A and B). In this study, no differences in all analyses were found between the WT strain and the complemented strain C-Δ*Hdac3*, so the results about C-Δ*Hdac3* were not mentioned in the text but presented in figures and tables. We further found that the ability of the WT to penetrate the insect cuticle did not differ from that of Δ*Hdac3* (Fig. 1C). However, the number of WT hyphal bodies in the hemocoel was 17-fold greater than Δ*Hdac3* (Fig. 1D).

### HDAC3 regulates ergosterol biosynthesis for oxidative stress tolerance during hemocoel colonization

We then investigated how HDAC3 regulated hemocoel colonization. First, we assayed the expression pattern of *Hdac3*. Compared to the penetrating hyphae (30 h after inoculation) on the cuticle of *G. mellonella* larvae, qRT-PCR analysis showed that the *Hdac3* expression was reduced 45-fold in the hyphal bodies in the larval hemocoel. The expression of *Hdac3* on cuticle was 4.5-fold higher than that in the mycelia grown in the nutrient-rich SDY medium (Saubraud dextrose broth supplemented with 1% of yeast extract) (Fig. 1E). Although the expression level of *Hdac3* was the lowest in the hyphal bodies, it was still expressed during hemocoel colonization [the quantification cycle of qRT-PCR reaction (Cq value) was around 30 when the complementary DNAs (cDNAs) were 4 ng (total RNA equivalents) in a 20 µL of qRT-PCR mixture]. We then tried to identify the target sites of HDAC3 by immunoblot analysis of the acetylation levels of eight lysine residues in the histone H3 and four in the histone H4. Compared to the WT strain, the acetylation level of histone H3 on lysine 56 (H3K56) was increased 2.6-fold in the mutant Δ*Hdac3*; no significant differences in acetylation level on other lysine residues in the histone H3 and H4 were found between the WT and Δ*Hdac3* (Fig. 1F; Fig. S2), suggesting that HDAC3 could catalyze the deacetylation of histone H3K56.

**Fig 2:**
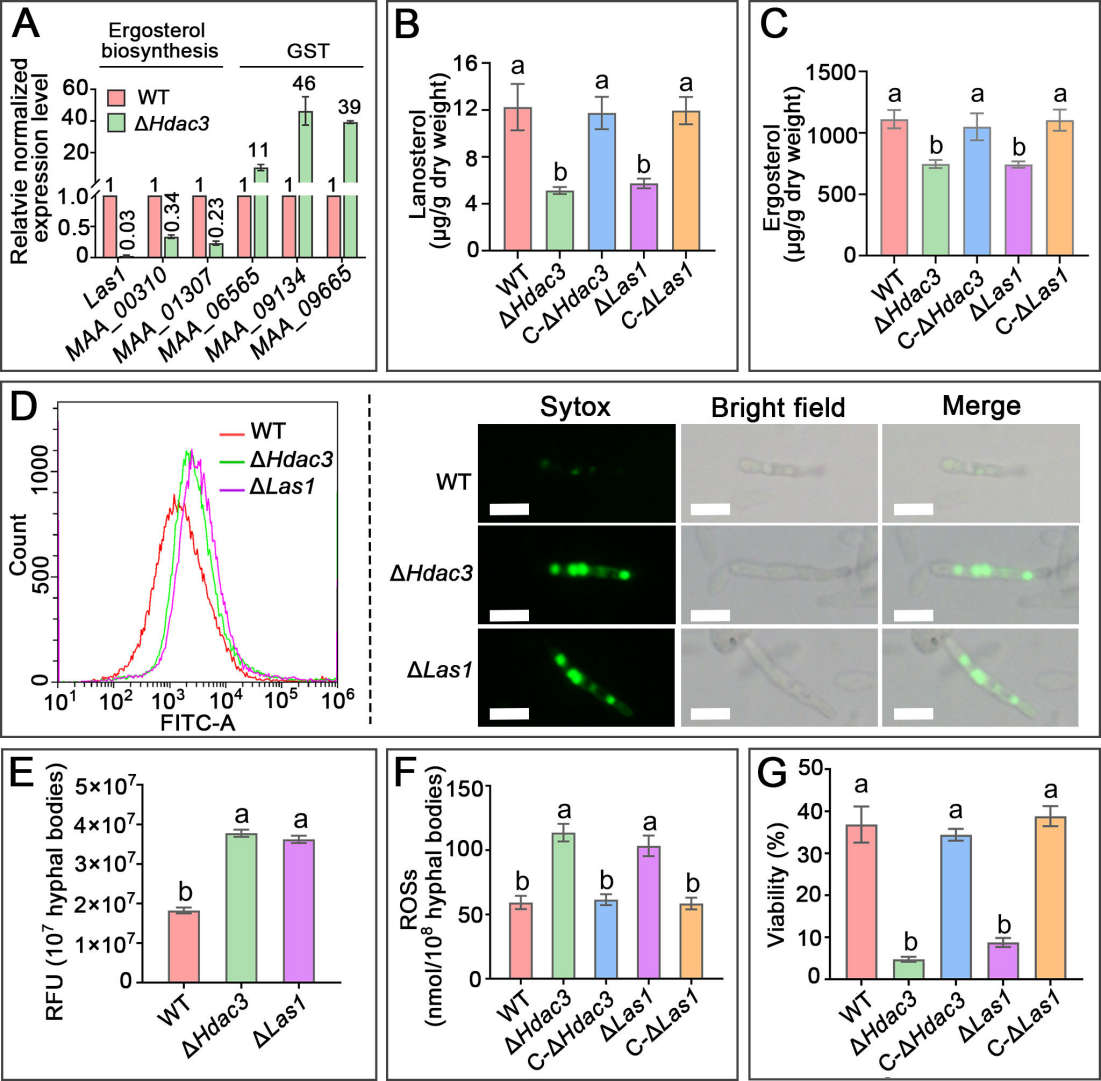
HDAC3 regulates ergosterol biosynthesis to maintain cell membrane integrity of hyphal bodies for oxidative stress tolerance. (**A**) qRT-PCR analysis of the expression of three genes in the ergosterol biosynthesis pathway and three GST genes in the hyphal bodies of the mutant Δ*Hdac3* and the WT strain. The values for a gene show the fold changes in the expression of the gene in the Δ*Hdac3* strain compared with the WT strain, which is set to 1. (**B**) Quantification of lanosterol and (**C**) ergosterol in the hyphal bodies of the WT, Δ*Hdac3,* and Δ*Las1*, and the complemented strains C-Δ*Hdac3* and C-Δ*Las1*. Data are expressed as the means ± SE. Values with different letters are significantly different (*P* < 0.05, Tukey’s test in one-way ANOVA). (**D**) Cell membrane integrity assays with Sytox Green staining. Left: flow cytometric assays of stained hyphal bodies. For each strain, 50,000 hyphal bodies were assayed. Right: representative hyphal bodies stained with Sytox Green. Scale bar: 5 µm. (**E**) Quantification of reactive oxygen species (ROS) levels in the hyphal bodies using the DCFH-DA, and (**F**) the OxiSelect *In Vitro* ROS/RNS Assay Kit. RFU: relative fluorescence unit. (**G**) The viability of hyphal bodies treated in an H_2_O_2_ solution (0.01%). Data are expressed as the means ± SE. Values with different letters are significantly different (*P* < 0.05, Tukey’s test in one-way ANOVA). All experiments were repeated three times.

We further used RNA-seq analysis to profile the differentially expressed genes (DEGs) between Δ*Hdac3* and WT hyphal bodies harvested from the hemocoel of infected *G. mellonella* larvae to identify genes regulated by HDAC3. There were 1,474 DEGs, with 1,039 genes upregulated and 435 genes downregulated in Δ*Hdac3*. HDAC3 did not regulate previously identified hemocoel colonization-related genes including the collagen-like gene *Mcl1* (13), cold shock protein *Crp1* (14), *Coh1,* and *Coh2* (2). However, RNA-seq and following qRT-PCR analysis showed that three antioxidant genes [glutathione S-transferases (GenBank accession numbers: MAA_06565, MAA_09134, and MAA_09665)] were upregulated over 10-fold in Δ*Hdac3*, and three genes (GenBank accession numbers: MAA_06587, MAA_01307, and MAA_00310) involved in ergosterol biosynthesis were downregulated (Fig. 2A). CUT and Tag (Cleavage Under Targets and Tagmentation) assays showed no differences between the WT and Δ*Hdac3* in the acetylation level on the histone H3 in the promoter regions of all six genes (Fig. S3A), suggesting that HDAC3 regulated their expression in an indirect manner. Two transcription factors (MAA_01460 and MAA_09524) were upregulated in Δ*Hdac3* (Fig. S3B), and the other two (MAA_10378 and MAA_11605) were downregulated (Fig. S3C). We then investigated whether HDAC3 regulated the GST and ergosterol synthesis genes by controlling these four transcription factors. However, no significant difference in the expression of the GST and ergosterol synthesis genes in hyphal bodies was found between the WT strain and the deletion mutants of the four transcription factor genes (Fig. S3D and E), and the virulence of the four deletion mutants did not significantly differ from the WT strain (Fig. S3F), suggesting that regulation of the GST genes and ergosterol synthesis genes by HDAC3 was not through controlling these four transcription factors.

Although it has not been documented that Sir2 regulators control the expression of GST genes during fungal tolerance to oxidative stress, it is known that Sir2 regulators regulate the antioxidant superoxide dismutases (SODs) for fungal oxidative stress tolerance (15–17), so we did not follow up on the regulation of antioxidant production. As it has been previously reported that ROSs are accumulated in ergosterol biosynthesis-impaired fungal cells (18), we instead investigated a possible new function of the Sir2 regulator HDAC3, that is, regulation of the expression of ergosterol biosynthesis for tolerance to oxidative stress from insects. Among the three ergosterol biosynthesis genes, the lanosterol synthase gene (GenBank accession number: MAA_06587, designed as *Las1*) was upstream of the other two genes and its expression was the most (33.3-fold) reduced by the deletion of *Hdac3*. Similar to the expression pattern of *Hdac3*, the expression level of *Las1* in the hyphal bodies in the larval hemocoel was 10- and 69-fold lower than the mycelia grown in the SDY medium and on the insect cuticle, respectively (Fig. 1E). Therefore, *Las1* was used as a representative of the three *Hdac3*-regulated ergosterol biosynthesis genes, and its deletion mutant was thus constructed (Fig. S1). The deletion mutant of *Las1* (Δ*Las1*) was complemented to produce the strain C-Δ*Las1* (Fig. S1D and E). No differences in all analyses were found between the WT and C-Δ*Las1*, so the results about C-Δ*Las1* were not mentioned in the text but presented in figures and tables.

Liquid chromatography-tandem mass spectrometry (LC-MS/MS) and high-performance liquid chromatography (HPLC) analysis showed that the content of lanosterol (ergosterol) in Δ*Hdac3* hyphal bodies was 2 (1.5)-fold lower than the WT, which also had a significantly higher amount of ergosterol and lanosterol than Δ*Las1* (*P* < 0.05), but no significant difference was found between Δ*Las1* and Δ*Hdac3* (Fig. 2B and C). Ergosterol is a major component in the cell membranes, contributing to membrane integrity and selective permeability (19). We thus compared the cell membrane integrity of hyphal bodies of the WT, Δ*Hdac3,* and Δ*Las1* by staining with Sytox Green, which can cross incomplete cell membrane and stain genomic DNA to give green fluorescence (20). Both flow cytometric assays and confocal microscopic observation showed that the fluorescence intensity of the stained hyphal bodies of Δ*Las1* and Δ*Hdac3* was greater than the WT; no obvious difference was found between Δ*Hdac3* and Δ*Las1* (Fig. 2D). We further investigated whether impairment in cell membrane integrity in Δ*Hdac3* and Δ*Las1* hyphal bodies resulted in reactive oxygen species (ROS) accumulation. Using the ROS probe DCFH-DA, the fluorescence intensity in the WT hyphal bodies was nearly twofold weaker than Δ*Hdac3* (*P* < 0.05), which was not significantly different from Δ*Las1* (Fig. 2E). Likewise, assays using a quantification kit also showed that the ROS amounts in the WT hyphal bodies were twofold lower than Δ*Hdac3* and Δ*Las1* (*P* < 0.05) (Fig. 2F). Following up, we conducted *in vitro* assays of the susceptibility of the hyphal bodies to ROSs by assaying their viability after 1-h incubation in H_2_O_2_-containing PBS (0.01%); compared to the WT, the viability of Δ*Hdac3* and Δ*Las1* hyphal bodies was reduced 7.7- and 4.2-fold (*P* < 0.05) (Fig. 2G).

**Fig 3:**
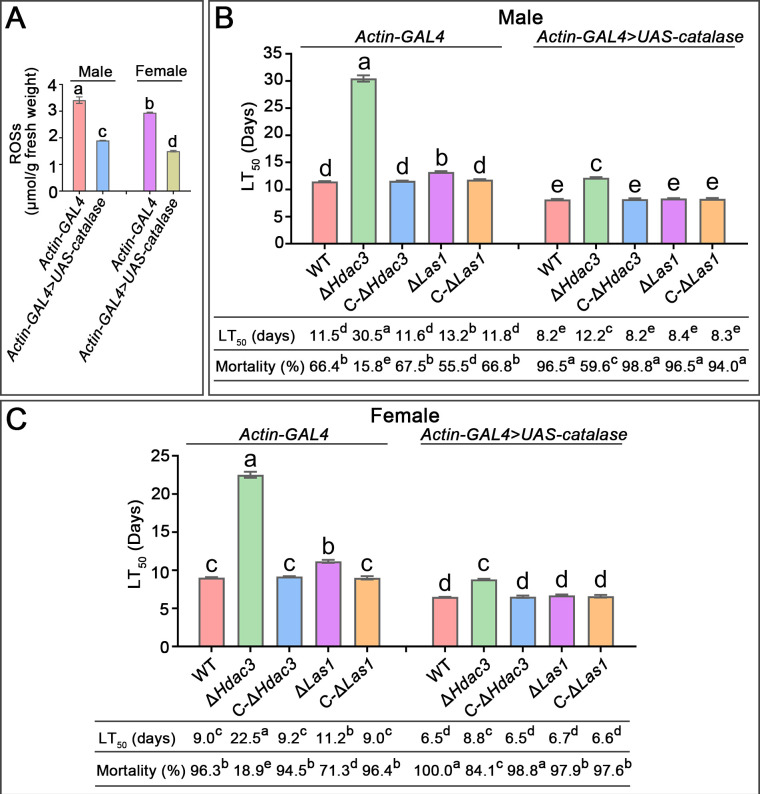
Pathogenicity against adults of the *D. melanogaster* line-deficient in ROS production (*Actin-GAL4 > UAS-catalase*) and the control line (*Actin-GAL4*). (**A**) Confirmation of the reduction in ROS production in the fly line *Actin-GAL4 > UAS-catalase*. (**B**) LT_50_ values against the males and (**C**) the females. Data are shown as the means ± SE. Values with different letters are significantly different (*P* < 0.05, Tukey’s test in one-way ANOVA) among all treatments. The mean LT_50_ values and mortalities at day 14 post-inoculation were shown. All experiments were repeated three times.

Similar to Δ*Hdac3*, bioassays against the *G. mellonella* larvae, via both topical application and direct hemocoel injection, showed that LT_50_ values of the WT were 1.2-fold lower than Δ*Las1*, which was in turn significantly lower than Δ*Hdac3* (*P* < 0.05) (Fig. 1A and B). We further confirmed the importance of the *Hdac3*-regulated ergosterol biosynthesis for tolerance to oxidative stress from insects by conducting bioassays using the *Drosophila melanogaster* line (*Actin-GAL4* > *UAS-Catalase*). Due to the overexpression of a catalase, the *Actin-GAL4* > *UAS-Catalase* line has a lower ROS level than its control line *Actin-GAL4* (21). We confirmed that the ROS level in the *Actin-GAL4* line was indeed significantly higher than *Actin-GAL4* > *UAS-Catalase* (*P* < 0.05) (Fig. 3A). The rate the WT strain killed both sexes of the *Actin-GAL4* > *UAS-Catalase* adults was nearly twofold faster than the *Actin-GAL4* (*P* < 0.05), suggesting that insect-derived oxidative stress is an important factor that limits fungal infection. Δ*Hdac3* was almost avirulent against both sexes of the *Actin-GAL4* flies, and at the experiment end (day 14 post-inoculation), it caused only 15.8% (18.9%) mortality in males (females), which was 4.2 (5.1)-fold less than the WT (*P* < 0.05). The rate Δ*Hdac3* killed *Actin-GAL4* > *UAS-Catalase* males (females) was 2.5 (2.6)-fold faster than *Actin-GAL4*, thus causing 3.8 (4.5)-fold higher mortality in males (females) of the *Actin-GAL4* > *UAS-Catalase* than those of *Actin-GAL4* (*P* < 0.05). The rates Δ*Las1* killed both sexes of the *Actin-GAL4* flies were significantly less than the WT but were significantly greater than Δ*Hdac3* (*P* < 0.05). Similar to Δ*Hdac3*, LT_50_ values of Δ*Las1* against *Actin-GAL4* flies were significantly higher than those against *Actin-GAL4* > *UAS-Catalase* (*P* < 0.05). The rates Δ*Las1* killed both sexes of the *Actin-GAL4* > *UAS-Catalase* flies were not significantly different from the WT but were significantly greater than Δ*Hdac3* (Fig. 3B and C).

In addition to oxidative stress, we also assayed whether HDAC3 regulates other factors that have been documented to be important for *M. robertsii* to colonize the insect hemocoel. Carbohydrate epitopes on hyphal body surfaces can be recognized by insects to trigger immune responses in the hemocoel (22). A series of lectins were thus used to compare the carbohydrate epitopes on hyphal bodies of the WT and Δ*Hdac3*. No differences in carbohydrate epitopes were found between the WT and Δ*Hdac3* hyphal bodies when the lectins GSL-II, PNA, Con A, and HPA were used. With the wheat germ agglutinin (WGA) targeting N-acetylglucosamine, the fluorescence intensity on Δ*Hdac3* cells was weaker than the WT (Fig. S4), but the recognition frequency of Δ*Hdac3* hyphal bodies by *G. mellonella* hemocytes was not different from the WT (Fig. S5A). In addition, no significant difference in activity and content of phenoloxidase was found between the *G. mellonella* larvae infected with the WT and Δ*Hdac3* (Fig. S5B). qRT-PCR analysis also showed that the expression levels of three antimicrobial peptides (gallerimycin, defensin, and cecropin) in the Δ*Hdac3*-infected insects were not significantly different from the insects infected by the WT (Fig. S5C). We further investigated whether HDAC3 regulated other factors for infection of insects based on the RNA-seq data described above; notably, four genes (GenBank accession numbers: MAA_02044, MAA_03396, MAA_06501, and MAA_07613), which are involved in peroxisome formation (KEGG pathway: maj04146), were downregulated in Δ*Hdac3* (Fig. S5D). We thus assayed the formation of peroxisomes in the WT and Δ*Hdac3* using the strains *WT-RFP-PTS1* and Δ*Hdac3-RFP-PTS1* that expressed the peroxisome maker protein RFP-PTS1 with the red fluorescent protein (RFP) fused with the peroxisome targeting signal type 1 (PTS1) from a long-chain acyl-CoA synthetase (GenBank accession number: MAA_01831). However, no difference in the number and size of peroxisomes was found between the WT and Δ*Hdac3* (Fig. S5E and F).

### HDAC3 regulates ergosterol biosynthesis for tolerance to the oxidative stress derived from plants

**Fig 4:**
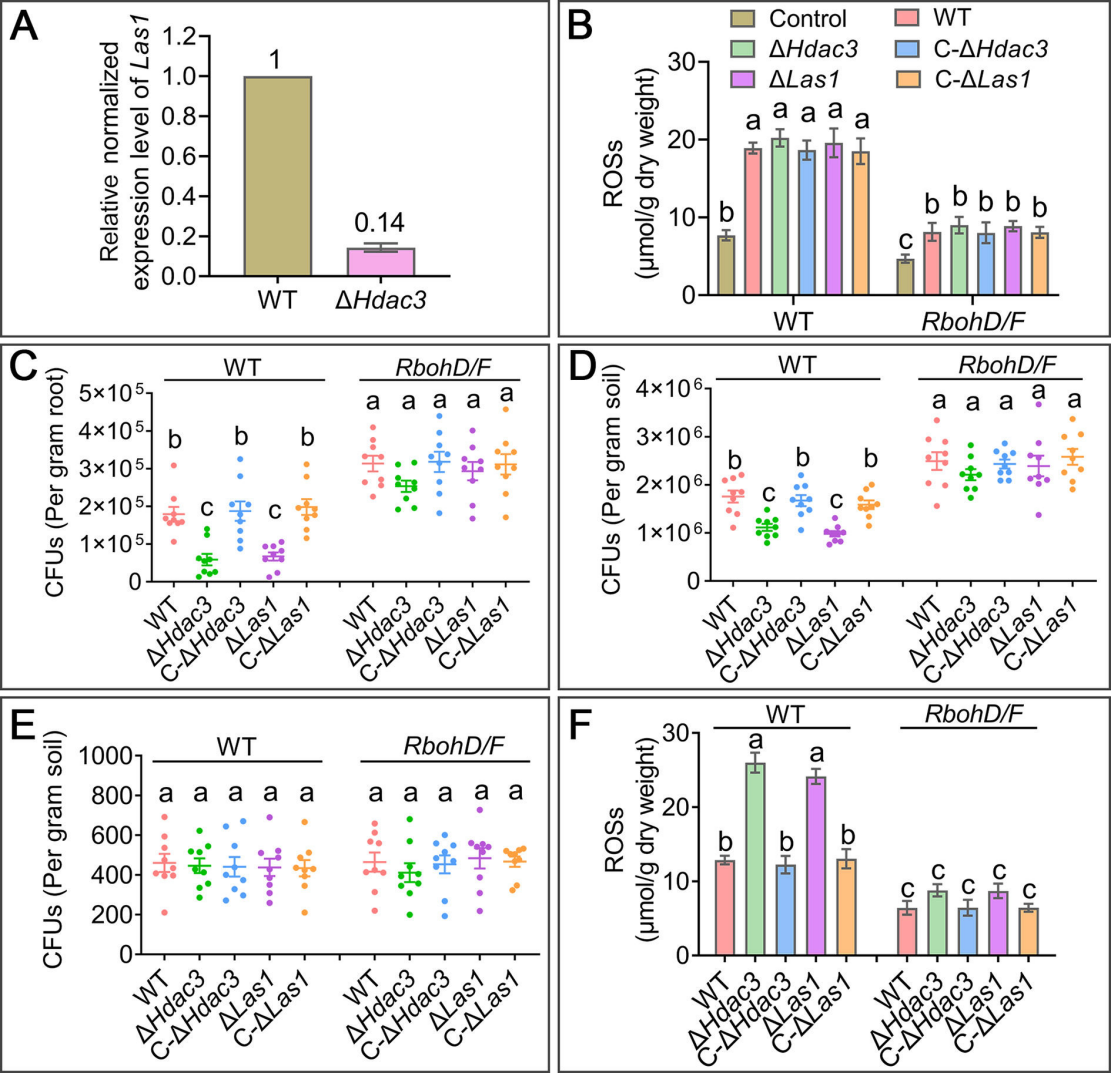
Colonization of roots and rhizosphere soil of the WT *A. thaliana* and the mutant *RbohD/F* that is deficient in ROS production. (**A**) qRT-PCR analysis of *Las1* expression in the WT and Δ*Hdac3* on the roots of the WT *A. thaliana* on the 1/2 MS medium. (**B**) ROS levels in roots of the WT and mutant plants inoculated with *M. robertsii*. Control: the plants not inoculated with fungi. (**C**) Colony-forming unit (CFU) counts in the roots, (**D**) rhizosphere soil, and (**E**) bulk soil at day 14 post-inoculation. (**F**) ROS levels in the mycelia colonizing *A. thaliana* grown on the 1/2 MS medium at day 5 post-inoculation. Data are shown as the means ± SE. Values with different letters are significantly different (*P* < 0.05, Tukey’s test in one-way ANOVA) among all treatments. All experiments were repeated three times.

Plant-associated fungi usually induce ROS burst in plant hosts, and they need to mitigate this oxidative stress to establish the plant and fungi associations (23). We thus investigated whether HDAC3-mediated regulation of ergosterol biosynthesis was important for tolerance to the plant-derived oxidative stress for successful development of symbiotic relationship between *M. robertsii* and plants. Compared to the WT hyphae colonizing roots of the WT *A. thaliana*, the expression of *Las1* was also reduced sevenfold in Δ*Hdac3* (Fig. 4A). We assayed the ability of the WT, Δ*Hdac3,* and Δ*Las1* to colonize the WT *A. thaliana* and its mutant *RbohD/F* with impaired production of ROSs due to deletion of two NADPH oxidase encoding genes (*RbohD* and *RbohF*) (24). We confirmed that the ROS level in the mutant *RbohD/F* was 1.6-fold lower than in the WT plants (*P* < 0.05). Inoculation of the WT strain induced the ROS level in the WT plants by 2.5-fold, which was 2.3-fold higher than that in the mutant *RbohD/F* inoculated with the WT strain (*P* < 0.05). No difference in the induction of ROS production by plants was found between the WT, Δ*Hdac3,* and Δ*Las1* (Fig. 4B).

**Fig 5:**
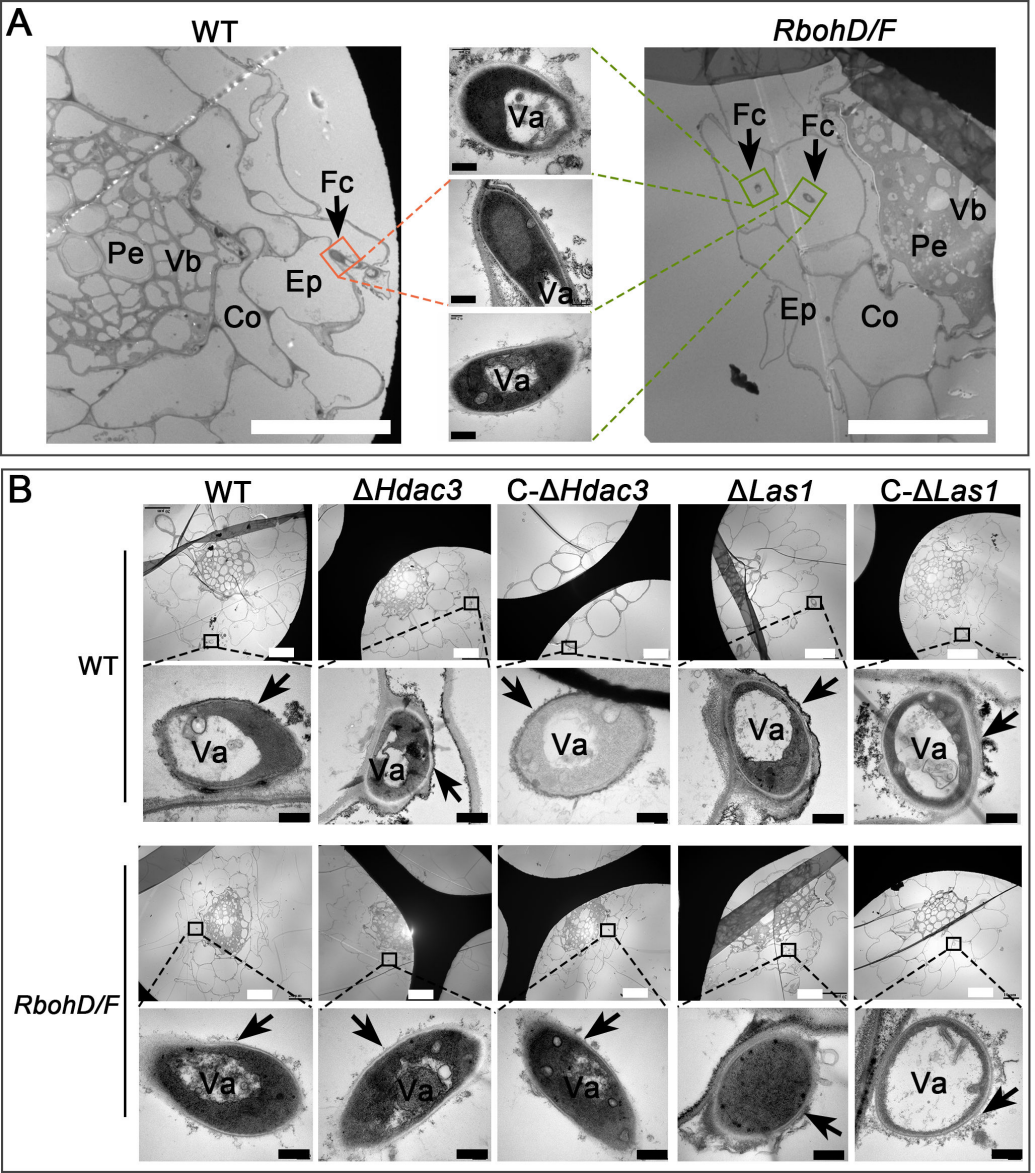
TEM analysis of colonization of *A. thaliana* roots and ROS deposition on the *Metarhizium* hyphae. (**A**) Root colonization by the WT strain at day 5 post-inoculation. (**B**) CeCl_3_-stained roots colonized with *Metarhizium* to show ROS deposition on the fungal cells at day 5 post-inoculation. Arrows: ROS deposition on the fungal cell walls. Scale bar: 20 µm (white) and 0.5 µm (black). Fc, fungal cells; Ep, epidermal cells; Co, cortex cells; Vb, vascular bundle; Pe, pericycle; and Va, vacuole in fungal cells. Enlarged insets: fungal cells colonizing rhizoplane or plant cells.

The colony-forming unit (CFU) counts of the WT *M. robertsii* from the WT *A. thaliana* roots were approximately threefold greater (approximately twofold in rhizosphere soil) than the mutants Δ*Hdac3* and Δ*Las1* (*P* < 0.05), and no significant difference was found between the two fungal mutants. In contrast, no significant difference in CFU counts in the roots and rhizosphere soil of the *A. thaliana* mutant *RbohD/F* was found between the WT, Δ*Hdac3,* and Δ*Las1* (Fig. 4C and D). The CFU counts of WT *M. robertsii* from the roots (rhizosphere soil) of the *A. thaliana* mutant *RbohD/F* were 1.7-fold (1.4-fold) higher than those from the WT plants (*P* < 0.05) (Fig. 4C and D). No significant differences in the CFU counts in bulk soil were found among all *M. robertsii* strains (Fig. 4E). The fresh weight, the length of primary roots, and the number of lateral roots of the plants colonized by the WT strain were not different from the mutant Δ*Hdac3* or Δ*Las1* (Fig. S6). Using TEM (transmission electronic microscopy), we further observed root colonization by the WT *M. robertsii*. On the WT *A. thaliana*, the hyphae were mostly seen on the root surface (rhizoplane) or between epidermal cells (that is extracellular colonization); however, hyphae were found inside the epidermal and cortex cells of the mutant *RbohD/F* (Fig. 5A), suggesting that plant-derived ROSs limited intracellular colonization by *M. robertsii*.

We further found that the WT mycelia colonizing the WT *A. thaliana* roots grown on the 1/2 MS medium contained twofold less amount of ROS than Δ*Hdac3* and Δ*Las1* (*P* < 0.05), and no significant difference was found between the two mutants, suggesting that more ROSs were accumulated in the mutant hyphae. For the hyphae colonizing roots of the mutant *RbohD/F*, no difference in ROS amount was found between the WT, Δ*Hdac3,* and Δ*Las1* (Fig. 4F). Analysis of localization of the deposition of electron-dense cerium perhydroxides using electron microscopy also showed that more ROSs were deposited on the surface of the Δ*Hdac3* and Δ*Las1* cells colonizing the WT *A. thaliana*, but no obvious difference was seen between the WT, Δ*Hdac3,* and Δ*Las1* cells colonizing the *A. thaliana* mutant *RbohD/F* (Fig. 5B).

### Regulation of ergosterol biosynthesis by HDAC3 is important for tolerance to abiotic oxidative stress

**Fig 6:**
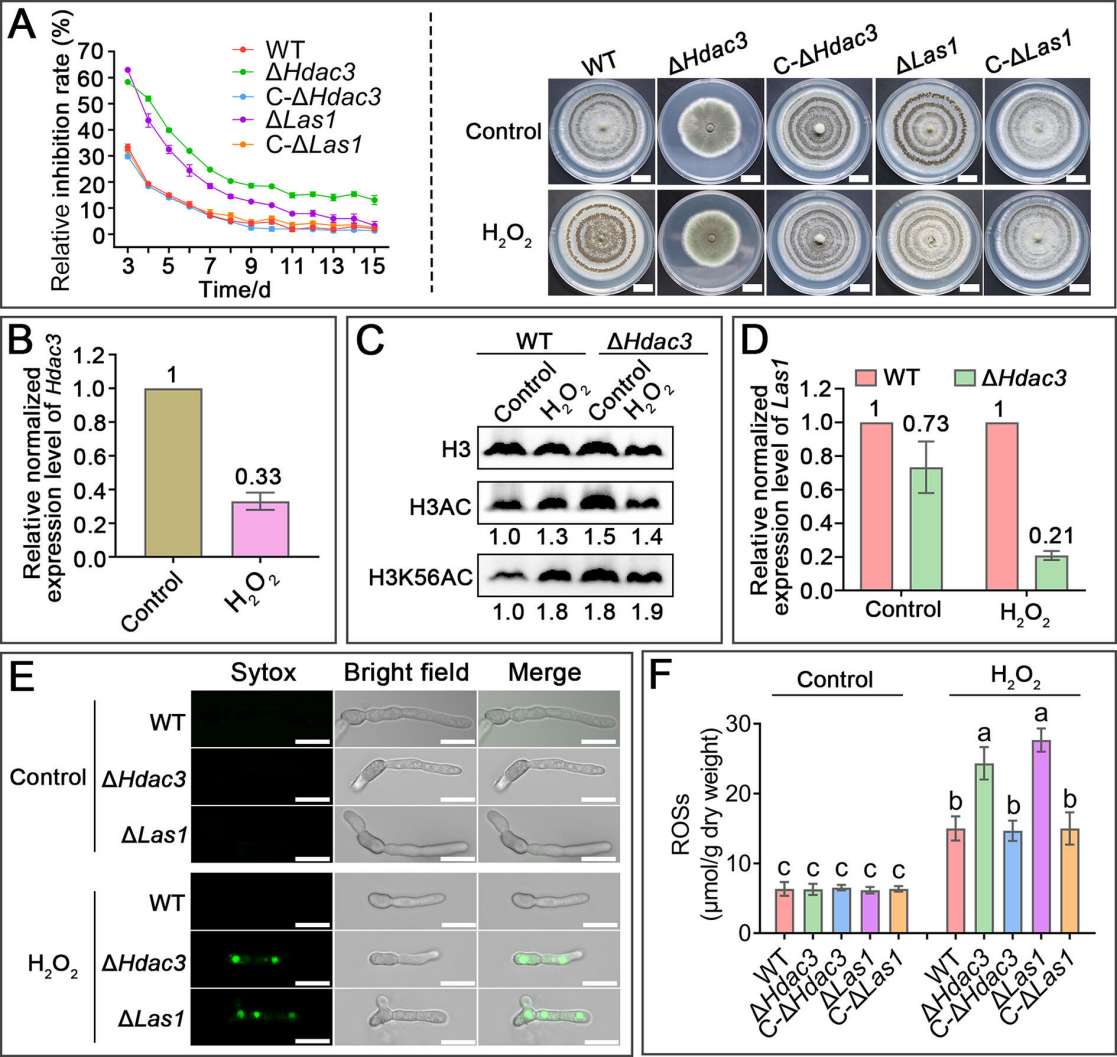
HDAC3 involved in abiotic oxidative stress tolerance. (**A**) Colony growth inhibition on PDA by H_2_O_2_. Left: relative growth inhibition rate over time on PDA containing 0.01% H_2_O_2_. Data are shown as the means ± SE. Right: representative pictures of colonies. Scale bar: 2 cm. (**B**) qRT-PCR analysis of *Hdac3* expression in the SDY medium without (control) or with H_2_O_2_. H_2_O_2_ (in panels B, **C, D, and F**): spores were cultured in the SDY at 26°C for 32 h, and H_2_O_2_ (0.01%) was then added for 4-h treatment. (**C**) Acetylation level in histone H3 and H3K56 in the WT and Δ*Hdac3* under oxidative stress. Numbers indicate the band intensity for acetylation level of histone H3 (lysine 56 in histone H3) relative to histone H3. Images shown are representatives of at least three independent experiments. (**D**) qRT-PCR analysis of *Las1* expression in Δ*Hdac3* and the WT, which is set to 1. (**E**) Representative hyphae stained with Sytox Green. Spores were cultured in the SDY for 16 h followed by 4-h treatment with H_2_O_2_ (0.01%). Scale bar: 10 µm. (**F**) ROS levels in the mycelia grown in the SDY without (control) or with H_2_O_2_. Data are shown as the means ± SE. Values with different letters are significantly different (*P* < 0.05, Tukey’s test in one-way ANOVA). All experiments were repeated three times.

In addition to the oxidative stress from the insect and plant hosts, we also assayed whether regulation of ergosterol biosynthesis by HDAC3 was also important for abiotic oxidative stress. We found that H_2_O_2_ supplemented in the PDA medium (0.01%) inhibited the growth rates of Δ*Hdac3* and Δ*Las1* to a significantly greater extent than the WT (*P* < 0.05) (Fig. 6A). But no difference in growth inhibition by high osmolarity, a stress encountered in the insect hemocoel, was found between the WT, Δ*Hdac3,* and Δ*Las1* (Fig. S7A). Likewise, no significant difference in growth inhibition by the cell wall-disturbing agent (Congo Red) was found between the WT, Δ*Hdac3,* and Δ*Las1* (Fig. S7B).

We then investigated how *Hdac3* responded to abiotic oxidative stress by assaying the impact of H_2_O_2_ on its expression and the acetylation level on H3K56. Supplementation of H_2_O_2_ in the SDY medium reduced the expression of *Hdac3* by threefold, thereby increasing the acetylation level on H3K56 by 1.8-fold. However, the inclusion of H_2_O_2_ had no significant impact on the acetylation level on H3K56 in the mutant Δ*Hdac3* (Fig. 6B and C). Therefore, oxidative stress-induced reduction in *Hdac3* expression largely determined oxidative stress-caused increase in the acetylation level on H3K56. In the SDY, no difference in *Las1* expression was found between the WT and Δ*Hdac3*, but in the H_2_O_2_-containing SDY, the expression level of *Las1* in WT was fivefold higher than Δ*Hdac3* (Fig. 6D). Consistent with the *Las1* expression pattern, Sytox Green staining assays showed that no difference in cell membrane integrity was found between the WT, Δ*Hdac3,* and Δ*Las1* when grown in the SDY, while in the H_2_O_2_-containing SDY, the cell membrane integrity of Δ*Hdac3* and Δ*Las1* was more severely damaged compared to the WT; no difference in cell membrane integrity was found between Δ*Hdac3* and Δ*Las1* (Fig. 6E). The ROS levels in the mycelia of Δ*Las1* and Δ*Hdac3* were the same as the WT in the SDY but were 1.7-fold higher than the WT in the H_2_O_2_-containing SDY (*P* < 0.05) (Fig. 6F).

## DISCUSSION

Oxidative stress is one of the most encountered stress types in any kind of fungal niche, and it can deleteriously and irreversibly impact cells, resulting in fungal degeneration or even death (25–27). Despite the mechanisms underlying fungal tolerance to oxidative stress have been extensively studied, understanding of regulatory mechanisms under oxidative stress remains limited. Currently, three regulators have been documented to regulate tolerance to oxidative stress in *Saccharomyces cerevisiae*: the HOG-MAPK, the transcription factors Yap1 and Skn7 (25). Skn7 is also involved in oxidative stress conferred by t-butyl-hydrogen peroxide in *M. robertsii* (12). Although Hog1-MAPK is conserved across the fungal kingdom (28), its involvement in the regulation of fungal tolerance to oxidative stress has diversified; Hog1-MAPK does not regulate oxidative stress tolerance in *M. robertsii* (29). Yap1 regulates oxidative stress tolerance by controlling the expression of the glutathione and thioredoxin system (25), but its homolog has not been characterized in *M. robertsii. M. robertsii* develops a parasitic relationship with insects and a mutually beneficial relationship with plants (30), but these two distinctive hosts both generate ROSs to limit the growth of *M. robertsii*. We showed that the histone H3 deacetylase HDAC3, a member of Sir2 family, is important for the fungus to survive the oxidative stress from both hosts and abiotic oxidative stress as well.

Sir2 family regulators are widely distributed from mammals to fungi (31) with diverse functions. Sir2 regulators have been characterized in several plant pathogenic fungi, in which Sir2 regulates oxidative stress tolerance by controlling the expression of SODs for the removal of plant-derived ROSs (15–17). In *M. robertsii*, HDAC3 does not regulate SOD expression, but GSTs. We further found that HDAC3 regulates ergosterol production to maintain cell membrane integrity, which is important for the fungus to survive oxidative stress. Similar to the cells of *Candida albicans* under oxidative stress (18), a reduction in ergosterol biosynthesis also resulted in ROS accumulation in the *M. robertsii* cells. Deletion of *Hdac3* had no impact on the acetylation level on the histone H3 in the promoter of the ergosterol biosynthesis genes (*Las1*) (Fig. S3A) and did not change the expression of the transcription factors (Upc2 and Ecm22) (Fig. S3G), which control the ergosterol biosynthesis pathway in other fungi (32); four *Hdac3*-regulated transcription factors did not control *Las1* expression (Fig. S3E). Similarly, three GSTs were upregulated in the mutant Δ*Hdac3* (Fig. 2A), but the deletion of *Hdac3* had no impact on the acetylation level on the histone H3 in the promoter regions of the three GST genes (Fig. S3A). Deleting the four *Hdac3*-regulated transcription factor genes also did not impact the expression of the GST genes (Fig. S3E). Therefore, *Hdac3* regulates ergosterol biosynthesis and GST production via other mechanisms that are to be identified in future work. The deletion mutant of *Las1* killed insects faster than the mutant Δ*Hdac3* (Fig. 1A, B, 3B, and C), suggesting that *Hdac3* also controls other virulence factors than those involved in oxidative stress. In contrast, no difference in colonization of rhizoplane and rhizosphere was found between Δ*Las1* and Δ*Hdac3* (Fig. 4C and D), indicating that *Hdac3* regulates plant colonization mainly via regulation of ergosterol biosynthesis. On regular PDA medium without oxidative stress, the growth rate of Δ*Hdac3* was slower than the WT, suggesting that HDAC3 is also involved in the regulation of key bioprocesses for fungal growth; investigation of such regulations in the future will facilitate a full understanding of HDAC3’s functions.

## MATERIALS AND METHODS

### Bacterial and fungal strains, insects, and plants

*M. robertsii* ARSEF23 and ARSEF2575 were obtained from the Agricultural Research Service Collection of Entomopathogenic Fungal Cultures (US Department of Agriculture). The deletion mutant of *Hdac3* in ARSEF2575 was previously reported (2). Vectors were constructed with *Escherichia coli* DH5α. *Agrobacterium tumefaciens* AGL1 was used for fungal transformations as previously described (33). All fungal strains and vectors used in this study are summarized in Table S1.

*G. mellonella* larvae were commercially purchased from Yuhui Biotechnology Co., Ltd (Tianjing, China). Two *D. melanogaster* lines (*Actin-GAL4* and *Actin-GAL4* > *UAS-Catalase*) were kindly provided by Prof. Jianhua Huang at Zhejiang University.

*A. thaliana* ecotype Columbia (Col-0) was obtained from the ABRC center (*Arabidopsis* Biological Resource Center) at Ohio State University (Columbus, OH, USA). The *A. thaliana RbohD/F* mutant was kindly provided by Dr. Kun Jiang at Zhejiang University.

### Gene deletion and complementation

Gene deletion and complementation were conducted as previously described (33). The flanking DNA fragments (~1,200 bp) of the open reading frame (ORF) of the gene to be deleted were cloned by PCR using the Phanta DNA polymerase (Vazyme, China), which were then inserted into the vector pPK2-Sur-GFP (33) to result in the deletion vector. All PCR products were confirmed by DNA sequencing. The primers used in this study are all presented in Table S2. To complement the deletion mutant of a gene, the fragment, containing its ORF and promoter region (~2 Kb) and terminator region (~300 bp), was amplified by PCR and then inserted into the vector pPK2-NTC-GFP (34). The resulting vector was transformed into the gene deletion mutant via *A. tumefaciens* to produce the complemented strain as previously described (33).

### Bioassays

Bioassays were conducted using *G. mellonella* larvae and *D. melanogaster* adults. For *G. mellonella* larvae, inoculations were performed either by immersion of larvae in a conidial suspension (3 × 10^7^ conidia/mL) (topical application) or via direct injection of 500 spores (in 5 µL of 0.01% Triton X-100) into the hemocoel (hemocoel injection) as previously described (20). For *D. melanogaster* adults, topical applications (2.5 × 10^4^ conidia/mL) were conducted as previously described (35). All bioassays were repeated three times with at least 45 insects per repeat.

The ability to penetrate the insect cuticle was assayed as previously described (36). The cuticle of *G. mellonella* larvae was prepared as previously described (2). To quantify hyphal bodies in the hemocoel of *G. mellonella* larvae*,* 10 µL of hemolymph was collected from an insect, and the number of hyphal bodies was then counted using a hemocytometer (Marienfeld, Germany).

### Assays of the acetylation level on lysine residues in histone H3 and H4

Immunoblot analysis of the acetylation levels of eight lysine residues in histone H3 and four in histone H4 was conducted as previously described (2). Anti-H3 and H4 antibodies were purchased from Merck Millipore (Germany). Antibodies against the acetylation of different lysine residues in histone H3 (H3K4, H3K9, H3K14, H3K18, H3K23, H3K27, and H3K56) and H4 (H4K5, H4K8, H4K12, and H4K16) were all purchased from Abclonal (China). The band intensity was quantified with Image J.

### Assays of colonization of roots and rhizosphere soil

Colonization of roots and rhizosphere soil of *A. thaliana* was assayed according to a previous protocol (3) with modifications. Briefly, one 10-day-old *A. thaliana* seedling was transplanted from 1/2 MS (Murashige and Skoog) plate into sterile soil in a nylon bag (100 mesh, diameter = 25 mm, and height = 80 mm), which was placed in the same soil in a flowerpot (diameter = 70 mm and height = 90 mm). The sterile soil contained three-quarters of nutrient soil (Shenzhibei, China) and one-quarter of vermiculite (Huakaiyinuo, China). The seedlings were cultivated in a growth chamber at 25°C with a photoperiod of 16 h of light/8 h of darkness. After 7 days, a conidial suspension (1 × 10^6^ conidia/mL) was injected into the rhizosphere of an *A. thaliana* plant (1 mL per plant) with a pipette. After 14 days, the rhizosphere soil and bulk soil (the soil outside of the nylon bag) were harvested for CFU counting on a *Metarhizium*-selective medium (37). To quantify root colonization, the plants were gently rinsed with sterile water to remove all soil and dried with sterile paper tissue. The roots were then weighed and placed into 1.5 mL tubes each containing 500 µL of 0.01% Triton X-100 and 0.5 g ceramic beads, which were then subjected to grinding with an automatic grinder (Jingxing, China). The resulting homogenates were plated onto the *Metarhizium-*selective medium to allow fungal growth. After 5 days, the CFUs were counted, and the number of CFUs per gram of root (fresh weight) or soil (dry weight) was then calculated. The experiments were repeated at least three times with three replicates per repeat.

### Assays of tolerance to abiotic stresses

Tolerance to abiotic stresses was assayed as previously described (29). PDA plates supplemented with H_2_O_2_ (0.01%), sorbitol (1.2 M), and Congo red (1 g/L) were used to produce oxidative stresses, osmotic stress, and cell wall disturbing stress, respectively. The relative inhibition of colony growth was calculated as (Dc − Dt)/Dc × 100%, where Dc and Ds represented the diameter of the colony with or without stress treatment (38). The experiments were repeated three times with three replicates per repeat.

To assay the ability of hyphal bodies from the insect hemocoel to tolerate oxidative stress, around 1 million hyphal bodies were treated for 1 h in H_2_O_2_-containing PBS (0.01%). The hyphal bodies were collected by centrifugation, which were then suspended with PBS (1 mL). The suspension was then evenly spread onto the *Metarhizium*-selective medium to allow fungal growth, and the viability of the hyphal bodies was determined by the number of CFU counts. The experiments were repeated three times.

### Assays of carbohydrate epitopes

Assays of the carbohydrate epitopes of hyphal bodies were performed as previously described (22) with some modifications. Hyphal bodies from the insect hemocoel were suspended in PBS, which were then mixed with fluorescent WGA (20 µg/mL), *Griffonia simplicifolia* lectin (GSL-II, 20 µg/mL), *Arachis hypogaea* (peanut) lectin (PNA, 60 µg/mL), Concanavalin A (Con A, 20 µg/mL), or *Helix pomatia* lectin (HPA, 20 µg/mL). All lectins were purchased from Thermo Fisher Scientific (USA). After 1-h incubation in darkness, hyphal bodies were washed with PBS three times and then subjected to flow cytometric assays. For each assay, 50,000 hyphal bodies were analyzed. The experiments were repeated three times.

### Assays of peroxisome formation

The peroxisome formation was assayed by observing RFP-labeled peroxisomes as previously described (39). The peroxisome targeting signal type 1 (PTS1) (Ala-Lys-Leu) of the long-chain acyl-CoA synthetase (GenBank accession number: MAA_01831) was predicted using the PTS1 Predictor (https://mendel.imp.ac.at/pts1/). The coding sequence of the fusion protein RFP-PTS1 with PTS1 fused to the C-terminus of RFP was constructed using PCR. The resulting PCR product was cloned downstream of the constitutive promoter *Ptef* in the vector pPK2-Bar-Ptef (2) to produce the vector (Bar-RFP-PTS1). The vector was then used for the transformation of the WT, Δ*Hdac3,* and C-Δ*Hdac3* to produce the strains *WT-RFP-PTS1*, Δ*Hdac3-RFP-PTS1,* and *C-*Δ*Hdac3-RFP-PTS1*, respectively. Hyphal bodies from the insect hemocoel were first stained with Calcofluor white, which were then subjected to peroxisome observation using the laser scanning confocal microscope (Olympus, Japan). The number and size (μm^2^ per peroxisome) of the peroxisomes were quantified by Image J.

### Assays of immune responses

Assays of the recognition of hyphal bodies by the hemocytes of *G. mellonella* larvae were conducted as previously described with some modifications (40). Hemocytes were placed into a Petri dish (diameter = 35 mm, 40,000 hemocytes per plate), which contained 2 mL of Grace’s insect medium (Thermo Fisher Scientific, USA). After 2 h of incubation at 28°C, 2,000 hyphal bodies were added into the Petri dish. After 1-h incubation at 28°C, recognition of hyphal bodies by the hemocytes was observed under an inverted microscope (Leica, Germany). The experiments were repeated three times.

To assay the impacts of fungal infection on phenoloxidase expression and activity, *G. mellonella* larvae were inoculated with *Metarhizium* via topical application (1 × 10^7^ conidia/mL). Three days after inoculation, the hemolymph was collected for assays of phenoloxidase expression with Western blotting analysis as previously described (41). Anti-phenoloxidase antibody was kindly provided by Prof. Erjun Lin at the Institute of Plant Physiology and Ecology CAS China. The phenoloxidase activity in the hemolymph was detected as previously described (2). The experiments were repeated three times. To analyze the expression levels of antimicrobial-encoding genes, total RNA was extracted from the fat bodies of *G. mellonella* larvae.

### RNA-seq and qRT-PCR analysis

Total RNA was extracted with the Trizol reagent (Agbio, China). RNA-seq analysis was performed by Novogene Technology (China), and the details of the RNA-seq analysis were previously described (2).

For qRT-PCR, ReverTra Ace qPCR RT Master Mix (Toyobo, Japan) was used to synthesize cDNA using total RNA. qRT-PCR was conducted using TOROIVD 5G qPCR Premix (Torovid, China). The genes *Gpd* and *Tef* were used as internal standards for *Metarhizium* as previously described (42). The 18S rRNA gene (GenBank accession number: AF286298) was used as an internal standard for *G. mellonella* (2). The relative expression level of a gene was determined using the 2^−ΔΔCt^ method (43). All qRT-PCR experiments were repeated three times.

### Quantification of lanosterol and ergosterol

Total sterol was extracted with a previously described protocol (44). The hemolymph of the insects inoculated with *M. robertsii* was centrifuged at 8,000 rpm for 1 min to collect the hyphal bodies. The hyphal bodies were washed with ddH_2_O three times to remove the residual hemolymph and then shaken on the vortex mixer (Damlab, China) for 1 min to break the insect hemocytes. The hyphal bodies were washed with ddH_2_O three times again and then dried with the lyophilizer (Labconco, USA), which (20 mg) was then ground together with 0.5 g ceramic beads and 500 µL of the hydrolysis buffer (8 g NaOH + 20 mL H_2_O + 180 mL ethanol) using the automatic grinder. The resulting homogenate was transferred to a new 15-mL tube and replenished with 4.5 mL of the hydrolysis buffer, which was then incubated at 85°C for 2 h for cell lysis. The mixture was then mixed with H_2_O (1 mL) and cyclohexane (5 mL), followed by 15-min shaking for sterol extraction. The mixture was then centrifuged at 4,500 rpm for 10 min, and the sterol-containing upper phase was transferred into a new tube, and the residual sterol in the lower phase was further extracted with cyclohexane (5 mL) two times. The sterol mixtures from the three extractions were pooled and dried with a lyophilizer, and residues were suspended with methanol (500 µL).

Quantification of lanosterol with LC-MS/MS was performed as previously described (45). Liquid chromatogram analysis was performed on a Nexera SCL-40 HPLC System (Shimadzu, Japan). Lanosterol was separated on the Hypersil GOLD C18 column (100 × 2.1 mm, 1.9 µm) with a mobile phase, containing 95% methanol and 5% ammonium acetate aqueous solution (50 mM) supplemented with 0.1% formic acid at a flow rate of 0.5 mL min^−1^. The mass spectrometric detection was conducted using the Sciex QTRAP 6500^+^ System (Sciex, USA). The mass spectrometric detection was optimized in the positive ion detection mode by multiple reaction monitoring. The ion transition for lanosterol (*m*/*z*) was 409.5 to 109.2.

Quantification of ergosterol with HPLC was conducted as previously described (46). Ergosterol was analyzed with the Agilent 1200 Infinity HPLC system (Agilent Technologies, USA) and detected at 283 nm UV. Ergosterol was separated on the SB-C18 column (4.6 × 250 mm, 5 µm) with a mobile phase of 95% methanol.

Pure lanosterol and ergosterol were purchased from Sigma-Aldrich (USA) and dissolved in methanol. A series of solutions of lanosterol (ranging from 10 ng/mL to 10 µg/mL) and ergosterol (ranging from 62.5 µg/mL to 1 mg/mL) were prepared for standard curve plotting. The standard curve of lanosterol or ergosterol was plotted with the concentration of lanosterol or ergosterol (*X* axis) and the peak areas obtained from the LC-MS/MS or HPLC analysis (*Y* axis). All the experiments were repeated three times.

### Sytox Green staining

Sytox Green (Thermo Fisher Scientific, USA) staining was conducted as previously described (20) to assay the integrity of the cell membrane of fungal cells. The hyphal bodies collected from the insect hemocoel were first stained and then subjected to observation with a laser scanning confocal microscope or analysis using a flow cytometer (Beckman Coulter, USA); in the flow cytometric analysis, 50,000 hyphal bodies were assayed for each treatment. To stain hyphae grown in the SDY medium, spores were inoculated into the medium in confocal dishes (Biosharp, China). After incubation at 26°C for 16 h, H_2_O_2_ was added to achieve a final concentration of 0.01% to provide oxidative stress. After 4-h treatment, the hyphae were stained with Sytox Green and observed by the laser scanning confocal microscope. All the experiments were repeated three times.

### ROS assaying

To assay ROSs in the hyphal bodies using DCFH-DA staining, the fungal cells (1 × 10^7^) collected from the insect hemocoel were stained with DCFH-DA (10 mΜ, Abbkine, China) for 30 min in darkness. The hyphal bodies were washed three times with PBS to remove the residual DCFH-DA and suspended with PBS (200 µL) for the detection of the fluorescence intensity by Multimode Plate Reader (Thermo Fisher Scientific, USA). This experiment was repeated three times.

To quantify ROSs in fungal cells, adults of *D. melanogaster* (whole insects) or *A. thaliana* roots, the samples were ground together with PBS (500 µL) and ceramic beads (0.5 g) using the automatic grinder for ROS extraction. The resulting homogenates were transferred to new 1.5 mL tubes and subjected to centrifugation at 12,000 rpm at 4°C for 10 min. The supernatants were then subjected to ROS quantification using the OxiSelect *In Vitro* ROS/RNS Assay Kit (Cell Biolabs, USA). This experiment was repeated three times.

To prepare fungal cells from the fungus-colonized *A. thaliana* roots grown on the 1/2 MS medium, the roots colonized with mycelia were cut off from the plants and then vigorously washed in the Triton X-100 solution (0.05%) for 5 min to separate the mycelia from the roots, and the mycelia were then collected by centrifugation for ROS quantification. To prepare the roots for ROS quantification, the mycelia on the roots were carefully scalped, which were then vigorously washed three times in ample Triton X-100 solution (0.05%) to remove the mycelia as much as possible. The cleaned and washed roots were then subjected to ROS quantification. It is expected that there are still mycelia in some epidermal and cortex cells of the cleaned and washed roots; however, compared to a large amount of root biomass, the residual mycelial biomass could be negligible. Therefore, the cleaned and washed roots were treated as roots only for ROS quantification in this study.

Observation of ROS deposition on fungal cells colonizing *A. thaliana* roots was conducted with transmission electron microscope as previously described (47). Briefly, 10-day-old *A. thaliana* seedlings were transplanted from the 1/2 MS plates with sucrose onto sucrose-free 1/2 MS plates. After 1-day culture, a conidial suspension (1 × 10^6^ conidia/mL) was applied to the *A. thaliana* roots. After co-culture for 5 days, the roots were stained for 1 h with a CeCl_3_ solution [5 mM in the 3-(N-morpholino)-propanesulfonic acid buffer (50 mM, pH = 7.2)]. The roots were then washed three times with PBS and prefixed in a glutaraldehyde solution (2.5%) overnight at 4°C. The roots were then fixed, dehydrated, embedded, polymerized, cut, and stained with uranyl acetate and lead citrate as previously described (48). Ultra-thin sections (70 nm) of the sample were cut by the Leica UC 6 microtome (Austria) with a diamond knife (Diatome, Switzerland). TEM observation was conducted on the Hitachi 7650 transmission electron microscope (Japan).

### CUT and Tag assays

CUT and Tag assays were performed using the Hyperactive Universal CUT & Tag Assay Kit (Vazyme, China). Fungal protoplasts were prepared from hyphal bodies using the VinoTaste Pro for Maturation (Novozymes, Denmark). The protoplasts were then washed three times with the STC buffer (1 M Sorbitol, 50 mM Tris, and 50 mM CaCl_2_, pH = 8.0) and subjected to CUT & Tag assays with the Anti-acetyl Histone H3 antibody. The Normal Rabbit IgG (Cell Signaling Technology, USA) was used as the negative control. The resulting DNA was then used as a template for qPCR analysis. The experiments were repeated three times.

## Data Availability

RNA-seq data were deposited in the GenBank database [accession number: PRJNA1064586 (SRR27544716, SRR27544717, SRR27544718, SRR27544719, SRR27544720, and SRR27544721)].

## SUPPLEMENTAL MATERIAL

Supplemental Material msystems.00953-24-s0001.pdf Supplemental figures and tables.

ASM does not own the copyrights to Supplemental Material that may be linked to, or accessed through, an article. The authors have granted ASM a non-exclusive, world-wide license to publish the Supplemental Material files. Please contact the corresponding author directly for reuse.
