# Supplementary material for: Histone deacetylase HDAC3 regulates ergosterol production for oxidative stress tolerance in the entomopathogenic and endophytic fungus Metarhizium robertsii
Source: mSystems. 2024 Sep 17;9(10):e00953-24. doi: 10.1128/msystems.00953-24 (PMC11494875; doi:10.1128/msystems.00953-24)
Supplement: Supplemental Material — Supplemental figures and tables. [file msystems.00953-24-s0001.pdf]

SUPPLEMENTAL MATERIAL

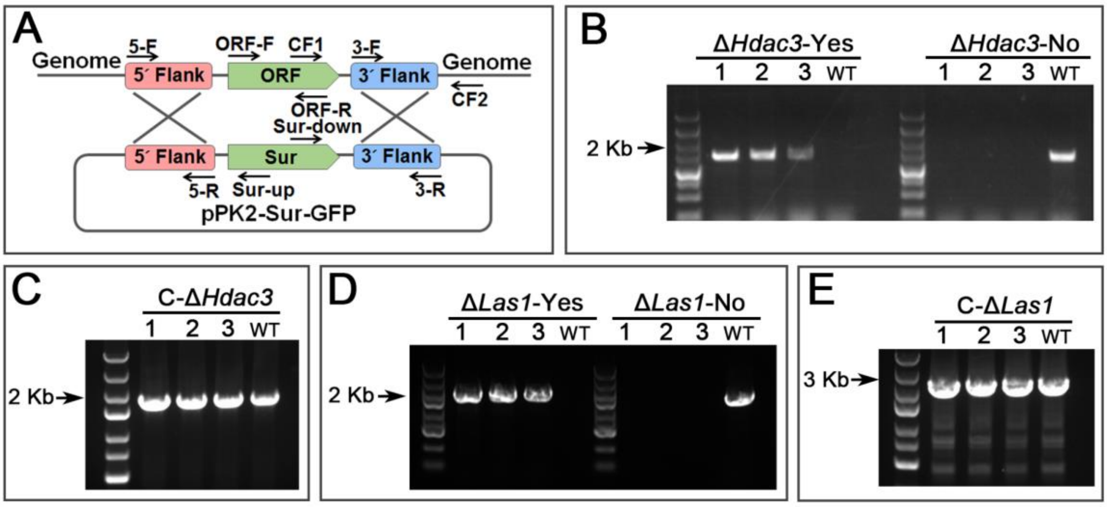

FIG S1 Construction of gene deletion mutants of *Hdac3* and *Las1* and their complementation. (**A**) A schematic diagram of gene deletion via homologous recombination using the herbicide resistance gene *Sur* as the selection marker. (**B**) Confirmation of the deletion of *Hdac3* in the *M. robertsii* ARSEF23 strain (WT). 1, 2, 3: three different deletion mutants. Yes: PCR conducted with the primers Sur-down and CF-2 to show the successful insertion of the selection marker gene. No: PCR conducted with the primers CF-1 and CF-2 to show the deletion of the ORF of a gene. The relative positions of primers are shown in A. (**C**) Complementation of the deletion mutant of *Hdac3*. 1, 2, 3: three different complemented strains. PCR conducted with primers ORF-F and ORF-R to show the successful complementation. (**D**) Confirmation of the deletion of *Las1* in *M. robertsii* ARSEF23. 1, 2, 3: three different deletion mutants. (**E**) Complementation of the deletion mutant of *Las1*. 1, 2, 3: three different complemented strains.

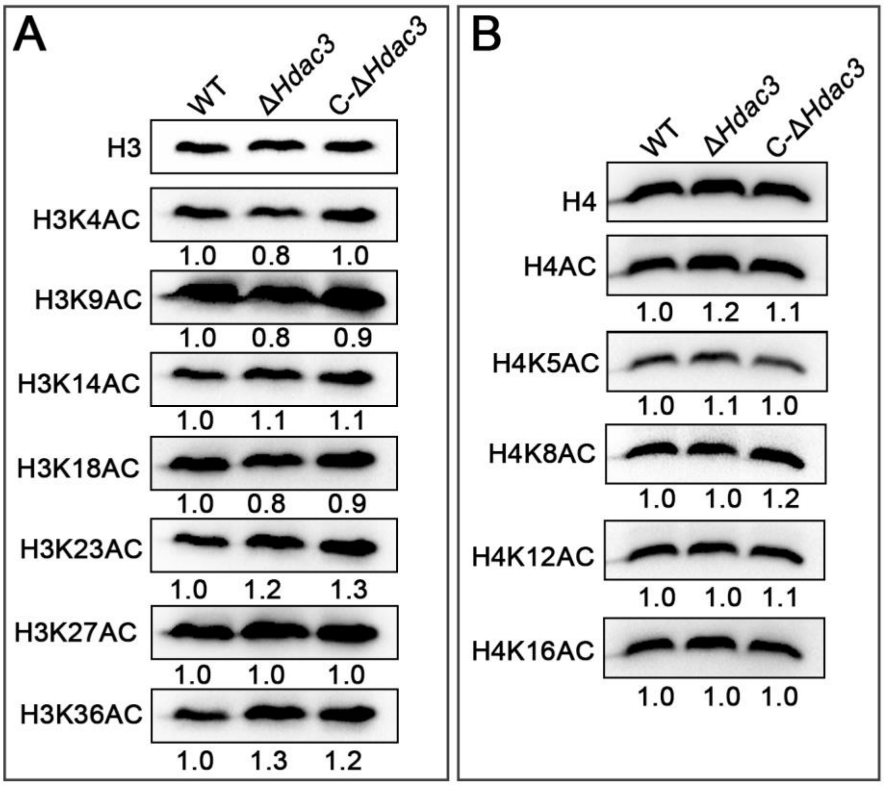

FIG S2 Immunoblot analysis of acetylation levels on lysine residues of histones. (**A**) Immunoblot analysis of acetylation levels on lysine residues of histone H3, and (**B**) histone H4. Images shown are representatives of at least three independent experiments. This figure is a supplement to Fig. 1F. Numbers indicate the band intensity for acetylation level of histone H3 (different lysine in histone H3) or H4 (different lysine in histone H4) relative to histone H3 or H4.

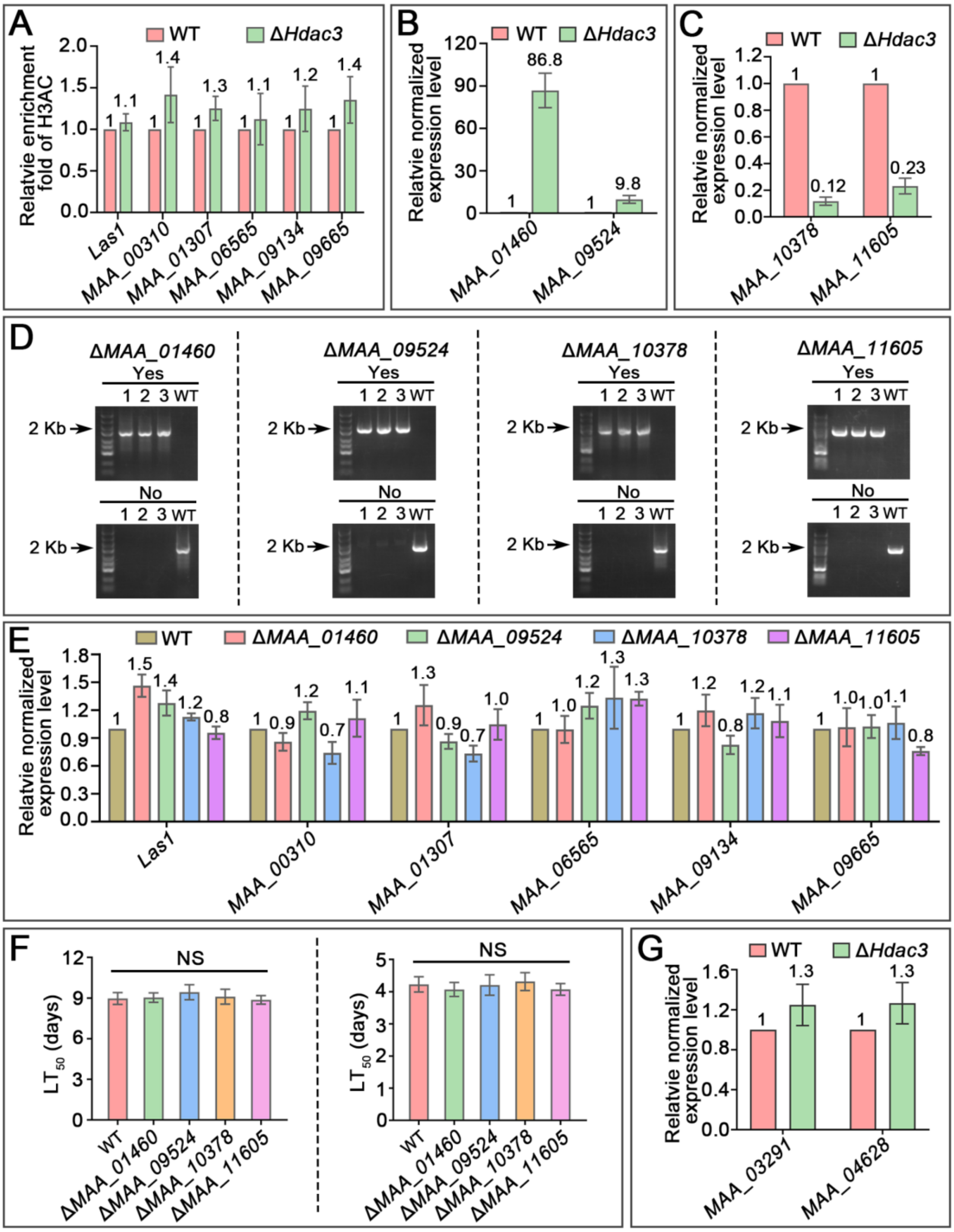

FIG S3 Regulation of three genes in the ergosterol biosynthesis pathway and three GST genes by HDAC3. (**A**) The relative enrichment of anti-H3Ac in the promoter region of a gene in Δ*Hdac3* versus the WT, which is set to 1. Data are expressed as the means ± SE. (**B**) qRT-PCR analysis of two up-regulated transcription factors and (**C**) two down-regulated transcription factors in the hyphal bodies of Δ*Hdac3* compared with the WT,

which is set to 1. (**D**) Construction of the deletion mutants (names are shown on the top) of four transcription factor genes. 1, 2, 3: three different deletion mutants. Yes: PCR conducted with the primers Sur-down and CF-2 to show the successful insertion of the selection marker gene. No: PCR conducted with the primers CF-1 and CF-2 to show the deletion of the ORF of a gene. (**E**) qRT-PCR analysis of the expression of three ergosterol biosynthesis genes and three GST genes in the hyphal bodies of the four transcription factor deletion mutants compared with the WT, which is set to 1. (**F**) LT_{50} values of the four transcription factor deletion mutants against the *G. mellonella* larvae via topical application (Left) and direct injection (Right). NS: not significant ($P > 0.05$, Tukey's test in one-way ANOVA). (**G**) qRT-PCR analysis of the expression of *Upc2* (GenBank accession number: MAA_03291) and *Ecm22* (GenBank accession number: MAA_04628) in the hyphal bodies of Δ*Hdac3* compared with the WT, which is set to 1.

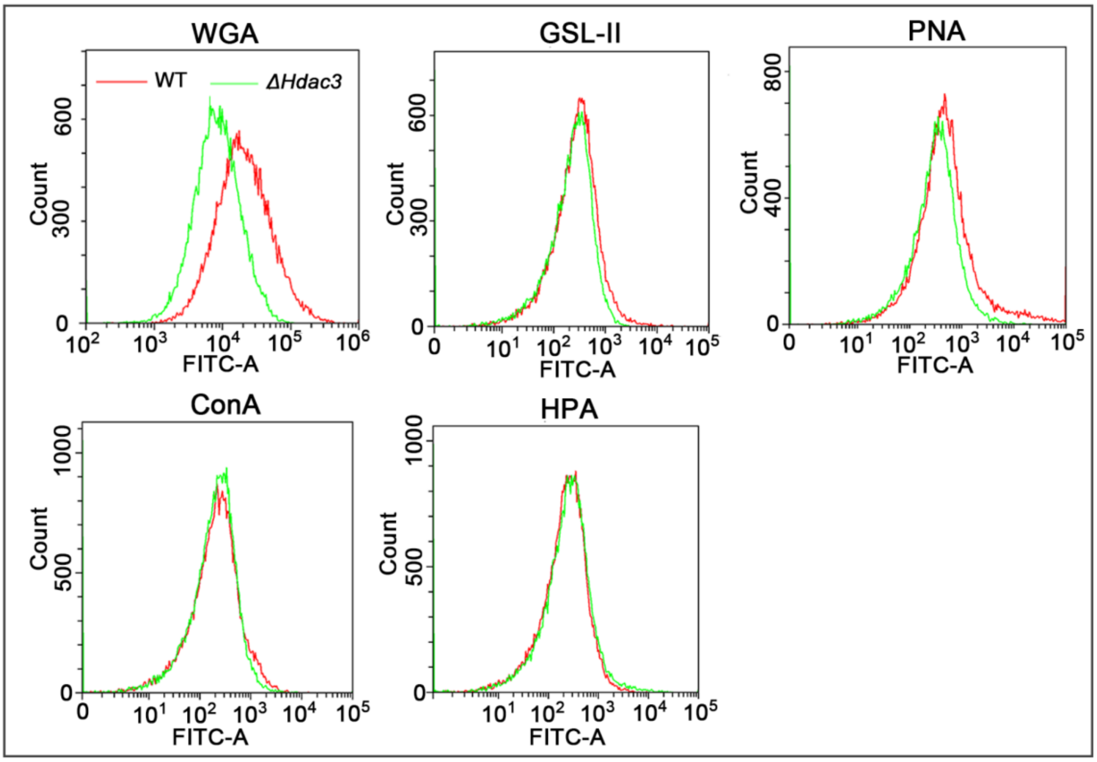

FIG S4 Flow cytometric analysis of hyphal bodies bound by lectins to show carbohydrate epitopes. WGA: wheat germ agglutinin; GSL-II: *Griffonia simplicifolia* lectin; PNA: *Arachis hypogaea* (peanut) lectin; Con A: Concanavalin A; HPA: *Helix pomatia* lectin. All experiments were repeated three times; shown in this figure are one of the repeats.

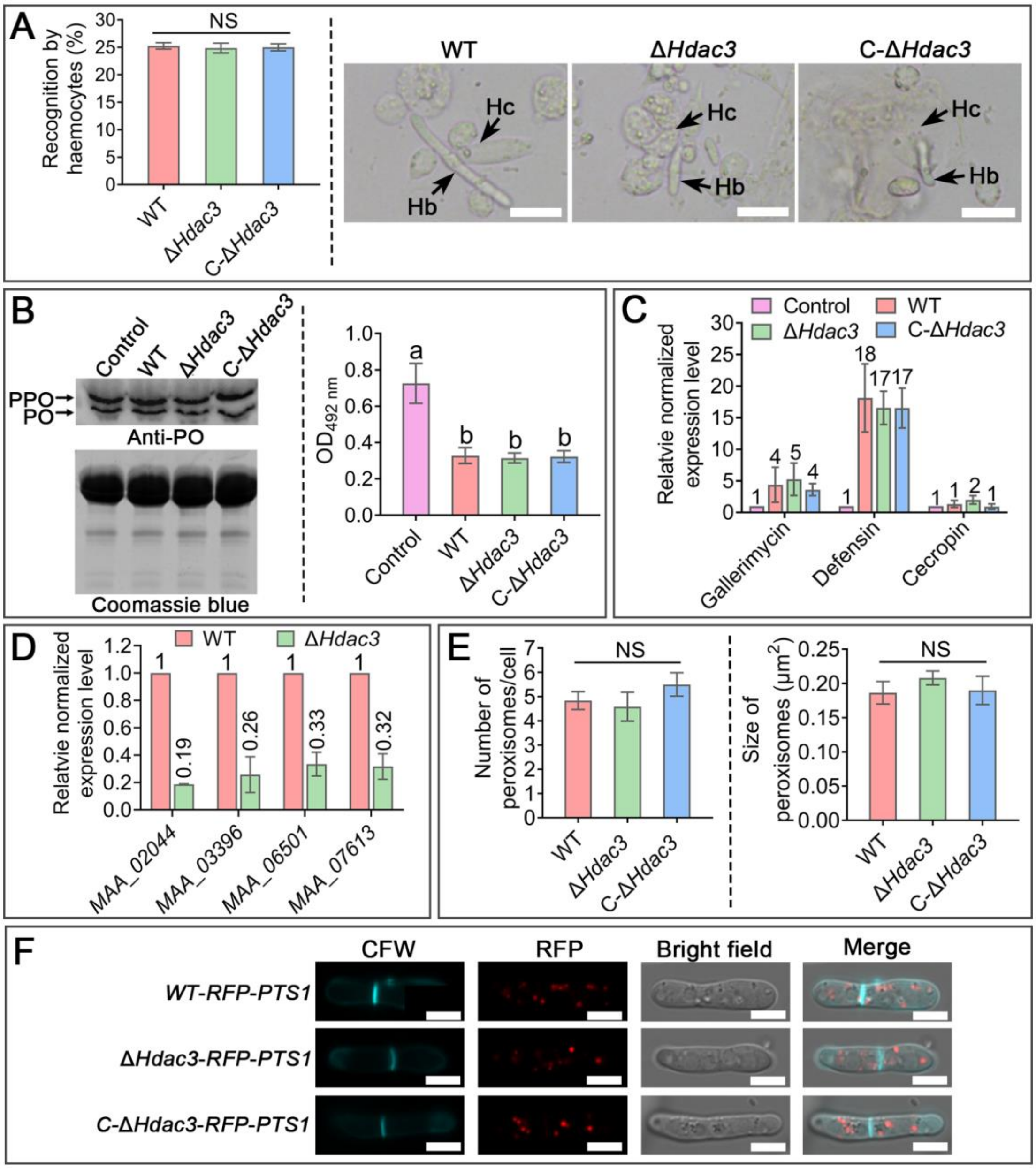

FIG S5 HDAC3 is not involved in the peroxisome formation, activation of encapsulation, and induction of phenoloxidase production and expression of antimicrobials by *G. mellonella* larvae. (**A**) Recognition of hyphal bodies by hemocytes. Left: quantification of recognition frequency. Data are expressed as the means ± SE. NS: not significant ($P > 0.05$, Tukey's test in one-way ANOVA). Right: representative images showing recognition of hyphal bodies by hemocytes. Hb: hyphal bodies; Hc: hemocytes. Scale bar: 20 μm. (**B**) The impact of deletion of *Hdac3* on the phenoloxidase

production and activity in insect hemocoel. Left: immunoblot analysis of phenoloxidase; Right: activity of phenoloxidase. Control: *G. mellonella* larvae treated with 0.01% Triton X-100. Data are expressed as the means ± SE. Values with different letters are significantly different ($P < 0.05$, Tukey's test in one-way ANOVA). (**C**) qRT-PCR analysis of genes encoding three antimicrobials. Control: larvae that were treated with 0.01% Triton X-100. (**D**) qRT-PCR analysis of the expression of four peroxisome formation-related genes in the hyphal bodies of Δ*Hdac3* and the WT, which is set to 1. (**E**) The number and size of the peroxisomes in the hyphal bodies. (**F**) Representative hyphal bodies of the strain *WT-RFP-PTS1*, Δ*Hdac3-RFP-PTS1* and *C-*Δ*Hdac3-RFP-PTS1*. CFW: hyphal bodies stained with the Calcofluor White. Scale bar: 5 μm. All experiments were repeated three times.

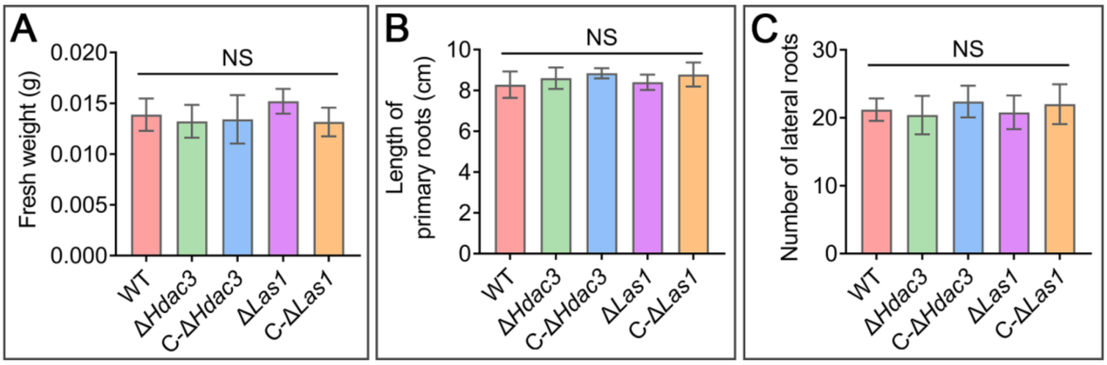

FIG S6 Growth of the WT *A. thaliana* plants colonized by *M. robertsii* on the 1/2 MS medium for 14 days. (**A**) Fresh weight. (**B**) Length of primary roots. (**C**) The number of lateral roots. WT: the wild-type strain; Δ*Hdac3*: the deletion mutant of *Hdac3*; Δ*Las1*: the deletion mutant of *Las1*; C-Δ*Hdac3*: the complemented strain of Δ*Hdac3*; C-Δ*Las1*: the complemented strain of Δ*Las1*. Data are expressed as the means ± SE. NS: not significant ($P > 0.05$, Tukey's test in one-way ANOVA). All experiments were repeated three times.

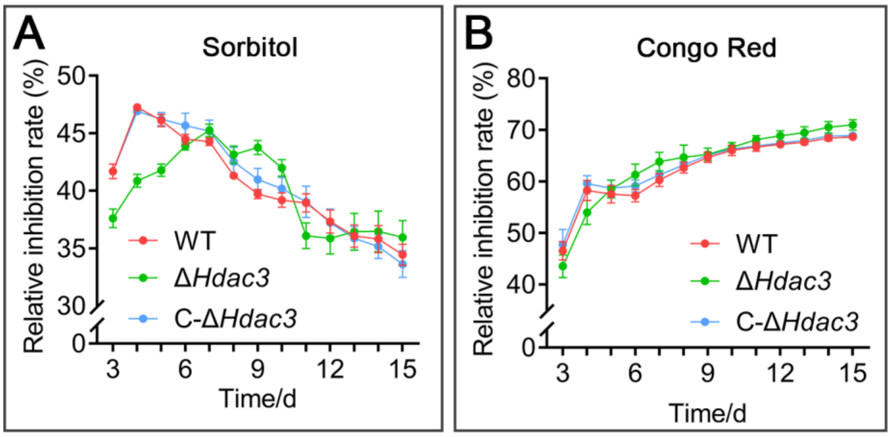

FIG S7 HDAC3 is not involved in tolerance to high osmolarity stress (1.2 M sorbitol) and the cell wall-disturbing agent Cong Red. (**A**) Colony growth inhibition by the high osmolarity stress (1.2 M sorbitol), and (**B**) Congo Red (1 g/L). Data are shown as the means ± SE. This experiment has been repeated three times with three replicates per repeat.

TABLE S1 Vectors and fungal strains used in this study.

Name	Description	Reference
**Plasmids**		
pPK2-Sur-GFP	Construction of gene deletion vectors	34
pPK2-NTC-GFP	Construction of gene complementation vectors	35
pPK2-Bar-Ptef	Construction of the vector that expressed the peroxisome maker protein RFP-PTS1	2
**Fungal strains**		
WT	The wild-type strain of *M. robertsii* ARSEF23 or ARSEF2575	
Δ*Hdac3*	The deletion mutant of *Hdac3* gene in ARSEF2575 or ARSEF2575	This study
C-Δ*Hdac3*	The complemented strain of the mutant Δ*Hdac3*-23	This study
Δ*Las1*	The deletion mutant of *Las1* gene in ARSEF23	This study
C-Δ*Las1*	The complemented strain of the mutant Δ*Las1*	This study
WT-RFP-PTS1	The WT strain that expressed RFP-PTS1 protein	This study
Δ*Hdac3-RFP-PTS1*	The mutant Δ*Hdac3* that expressed RFP-PTS1 protein	This study
*C-*Δ*Hdac3-RFP-PTS1*	The complemented strain of the mutant Δ*Las1* that expressed RFP-PTS1 protein	This study

TABLE S2 Primers used in this study

Primer name	Sequence	Usage
ΔHdac3-5-F	GCTCTAGAGAGTGCCATGAAGTTGTTACA	Deletion of *Hdac3*
ΔHdac3-5-R	CGGAATTCGGAGCAGGCGAGGTAAAGA	
ΔHdac3-3-F	GGACTAGTATAGAGAGCGGGAGGCTCCT	
ΔHdac3-3-R	AAAAGTACTCCTCTTATTTATAACACGCGA	
ΔHdac3-CF1	CCACAACCCTACTGCGTCTC	Confirmation of the deletion of *Hdac3*
ΔHdac3-CF2	TTCCATGTTCAGAAGCCCC	
Sur-down	ATCCAATGCACTCCTGTGGC	Confirmation of the deletion of *Hdac3* and *Las1*
C-ΔHdac3-F	CTAGCTAGCGAGTGGGAGGTGATTTTGATG	Complementation of *Hdac3* deletion mutant
C-ΔHdac3-R	AAAAGTACTTCCCACCGACCACGGTTAC	
Hdac3-F	ATGGAAGAAATTACCTCGAGTCC	Confirmation of the complementation of *Hdac3* deletion mutant
Hdac3-R	TCAAGAGACATCAATCAGGTTGC	
ΔLas1-5-F	GGACTAGTCATTAGATCGTGTGCGACAGTG	Deletion of *Las1*
ΔLas1-5-R	GAAGATCTGCCTTGGTGTGTATTGCTGTT	
ΔLas1-3-F	GGACTAGTGTCCTGGGGAGGACGACTT	
ΔLas1-3-R	TCCCCCGGGCTCTTTTTTTTTCTAGGTTCC TTT	
ΔLas1-CF1	TAGCGTGGCTAGTCAGACAGC	Confirmation of the deletion of *Las1*
ΔLas1-CF2	CTTGACCACCGAGTAAGAACTGA	
C-ΔLas1-F	GCTCTAGACAAAGATTGACAGAAGACAAA GAAG	Complementation of *Las1* deletion mutant
C-ΔLas1-R	GCTCTAGACGTTTGTCGTAGCTGGCAT	
Las1-F	ACATCTGCCTCAACTATGCCG	Confirmation of the complementation of *Las1* deletion mutant
Las1-R	CTACTGTTTCTGCAATTCGCAG	
RT-Gpd-F	GACTGCCCGCATTGAGAAG	qRT-PCR for *Gpd*
RT-Gpd-R	AGATGGAGGAGTTGGTGTTG	
RT-Tef-F	CTGGTACAAGGGTTGGGAGA	qRT-PCR for *Tef*
RT-Tef-R	TACACATCCTGGAGGGGAAG	
RT-Hdac3-F	ACACTCACCAAGGCAGGCAAGA	qRT-PCR for *Hdac3*
RT-Hdac3-R	AGACGGCAGCGATACCACCAA	
RT-Las1-F	CCAAGCGAGGCCCTAGATAA	qRT-PCR for *Las1*
RT-Las1-R	AACGTAGCGGATCATCTCGT	
RT-18S-F	CGCGCTACACTGAAGGAATC	qRT-PCR for 18S rRNA
RT-18S-R	AAGGGCAGGGACGTAATCAA	

RT-Gal-F	AAGATCGCTTTCATAGTCGCA	qRT-PCR for gallerimycin
RT-Gal-R	CTCGTAAAATACACATCCGGGG	
RT-Def-F	CGTCACCACAAAATGCTGTACA	qRT-PCR for defensin
RT-Def-R	CTACTCCTCGCACCAACAA	
RT-Cec-F	CGCGTTGGAAGGTGTTTAAA	qRT-PCR for cecropin
RT-Cec-R	TGATAGTAGATGCTTGACCCACA	
RT-0310-F	CAACTACGGATCCCTTTCCA	qRT-PCR for *MAA_00310*
RT-0310-R	GGCGATGTCTCCAAAGGTTA	
RT-1307-F	GCCGAGATACTCGACTCCTG	qRT-PCR for *MAA_01307*
RT-1307-R	CCCCATATCACCCTTACGTG	
RT-3291-F	GATCTACCACGTTGCCAACC	qRT-PCR for *MAA_03291*
RT-3291-R	GGAGAAGGTACCAATGGCCT	
RT-4628-F	ACGGCGTTTTCTGGTTTCTC	qRT-PCR for *MAA_04628*
RT-4628-R	ACGTATTTGAGCCATCGTGC	
RT-6565-F	GTTCCTCTACTTCCAGGCGT	qRT-PCR for *MAA_06565*
RT-6565-R	GGACACCTTCTTCTCCGACA	
RT-9134-F	GAAGCGTACAAACCGTCGAA	qRT-PCR for *MAA_09134*
RT-9134-R	TGAAGCAGTGCCTAGTGTCA	
RT-9665-F	CCCGAATACCTGTCAGACGT	qRT-PCR for *MAA_09665*
RT-9665-R	GACGTTGGCCCATACAATCC	
C&T-Las1-F	GGCGTGCACGTAGCACACA	CUT&Tag analysis of *Las1*
C&T-Las1-R	CGGACTGCAATAGAACAAATACTC	
C&T-0310-F	AAGTCAGCATTGAAGTTTAAAGGTT	CUT&Tag analysis of *MAA_00310*
C&T-0310-R	AGGTCCAAGTGTAAAAACGAGTACC	
C&T-1307-F	CCCTTTGCTGACAACTGTATCAC	CUT&Tag analysis of *MAA_01307*
C&T-1307-R	ATACAATGCAACGACGGCG	
C&T-6565-F	GGTTCTTACATCACGTCTGGTACA	CUT&Tag analysis of *MAA_06565*
C&T-6565-R	TTTTAATTTTGCAACATGCCACCTG	
C&T-9134-F	TACATGATCTCAGCGAAATAGCAA	CUT&Tag analysis of *MAA_09134*
C&T-9134-R	TTTTAATGACGGTAGACCCGAA	
C&T-9665-F	TTGGATCAATACAATACTCTTCGC	CUT&Tag analysis of

		MAA_09665
C&T-9665-R	CTGAGAGGCATTCACGCATATT	
RFP-PTS1-F	CGGGATCCATGGCCTCCTCCGAGGAC	Expression of the peroxisome maker protein RFP-PTS1
RFP-PTS1-R	GCGATATCTTACAGCTTAGCGGCGCCGGTG GAGTG	
